# Inhibition by Thyroid Hormones of Cell Migration Activated by IGF-1 and MCP-1 in THP-1 Monocytes: Focus on Signal Transduction Events Proximal to Integrin αvβ3

**DOI:** 10.3389/fcell.2021.651492

**Published:** 2021-04-08

**Authors:** Elena Candelotti, Roberto De Luca, Roberto Megna, Mariangela Maiolo, Paolo De Vito, Fabio Gionfra, Zulema Antonia Percario, Monica Borgatti, Roberto Gambari, Paul J. Davis, Hung-Yun Lin, Fabio Polticelli, Tiziana Persichini, Marco Colasanti, Elisabetta Affabris, Jens Z. Pedersen, Sandra Incerpi

**Affiliations:** ^1^Department of Science, Roma Tre University, Rome, Italy; ^2^Beth Israel Deaconess Medical Center, Harvard Medical School, Boston, MA, United States; ^3^Department of Biology, Tor Vergata University, Rome, Italy; ^4^Department of Life Sciences and Biotechnology, University of Ferrara, Ferrara, Italy; ^5^Department of Medicine, Albany Medical College, Albany, NY, United States; ^6^Pharmaceutical Research Institute, Albany College of Pharmacy and Health Sciences, Albany, NY, United States; ^7^Taipei Cancer Center, Taipei Medical University, Taipei, Taiwan; ^8^Graduate Institute of Cancer Biology and Drug Discovery, College of Medical Science and Technology, Taipei Medical University, Taipei, Taiwan; ^9^Traditional Herbal Medicine Research Center of Taipei Medical University Hospital, Taipei Medical University, Taipei, Taiwan; ^10^TMU Research Center of Cancer Translational Medicine, Taipei Medical University, Taipei, Taiwan

**Keywords:** PI3-kinase and ERK1/2 signaling pathways, nitric oxide, cytokine, STAT-1, molecular docking, reactive oxygen species, baicalein, tetrac

## Abstract

Interaction between thyroid hormones and the immune system is reported in the literature. Thyroid hormones, thyroxine, T_4_, but also T_3_, act non-genomically through mechanisms that involve a plasma membrane receptor αvβ3 integrin, a co-receptor for insulin-like growth factor-1 (IGF-1). Previous data from our laboratory show a crosstalk between thyroid hormones and IGF-1 because thyroid hormones inhibit the IGF-1-stimulated glucose uptake and cell proliferation in L-6 myoblasts, and the effects are mediated by integrin αvβ3. IGF-1 also behaves as a chemokine, being an important factor for tissue regeneration after damage. In the present study, using THP-1 human leukemic monocytes, expressing αvβ3 integrin in their cell membrane, we focused on the crosstalk between thyroid hormones and either IGF-1 or monocyte chemoattractant protein-1 (MCP-1), studying cell migration and proliferation stimulated by the two chemokines, and the role of αvβ3 integrin, using inhibitors of αvβ3 integrin and downstream pathways. Our results show that IGF-1 is a potent chemoattractant in THP-1 monocytes, stimulating cell migration, and thyroid hormone inhibits the effect through αvβ3 integrin. Thyroid hormone also inhibits IGF-1-stimulated cell proliferation through αvβ3 integrin, an example of a crosstalk between genomic and non-genomic effects. We also studied the effects of thyroid hormone on cell migration and proliferation induced by MCP-1, together with the pathways involved, by a pharmacological approach and docking simulation. Our findings show a different downstream signaling for IGF-1 and MCP-1 in THP-1 monocytes mediated by the plasma membrane receptor of thyroid hormones, integrin αvβ3.

## Introduction

Thyroid hormones 3,5,3′-triiodo-L-thyronine (T_3_) and L-thyroxine (T_4_) give rise to a wide range of effects on metabolism, growth, and development (Yen, 2001). The major form of thyroid hormone secreted from the thyroid gland is T_4_, whereas T_3_, the true hormone, is produced mainly in target tissues by deiodination of T_4_ (Bianco et al., 2002). The effects are known to be mediated by the binding of T_3_ to specific receptor proteins that may translocate to the cell nucleus where they regulate gene expression, and they require a period of time for protein synthesis and the biological response to manifest (Feng et al., 2000; Yen, 2001).

Non-genomic or extranuclear actions of thyroid hormone are initiated at the plasma membrane or in the cytoplasm and do not depend primarily on the interaction of the hormone with classical nuclear receptors (TRs). Non-genomic mechanisms of thyroid hormone action rely upon transduction of the hormone signal by kinases such as mitogen-activated protein kinase (MAPK) (Lin et al., 1999a; D’Arezzo et al., 2004; Lei et al., 2004, 2008; Cao et al., 2005; Davis et al., 2005; Cohen et al., 2011) or phosphoinositide 3-kinase (PI3K) (Bergh et al., 2005; Hiroi et al., 2006; Incerpi et al., 2014; Davis et al., 2016) that are cytoplasmic in location, but once they are activated may move to other intracellular compartments.

A crosstalk between the thyroid hormone-activated MAPK pathway and the signal transducer and activator of transcription (STAT) proteins has been reported, and this is important for the potentiation by thyroid hormone in the physiological concentration range of the actions of interferon-γ (IFN-γ) and epidermal growth factor (EGF) in HeLa cells that contain no nuclear receptor of thyroid hormone. Thyroid hormone causes tyrosine phosphorylation and nuclear translocation of STAT-1α by a non-genomic mechanism. In addition to this, thyroid hormones are able to modulate the activity of growth factors such as EGF and transforming growth factor-α (TGF-α) (Lin et al., 1999b; Shih et al., 2004).

The long-searched plasma membrane receptor for thyroid hormone is an integral transmembrane protein: the integrin αvβ3 (Bergh et al., 2005). The interaction of the integrin with thyroid hormone elicits a complex array of cellular events, and only some of them have been identified to date, leading to angiogenesis and tumor cell proliferation (Bergh et al., 2005; Davis et al., 2014, 2016; Mousa et al., 2018; Gionfra et al., 2019). The integrin αvβ3 is involved in several diseases (Berghoff et al., 2014; Bi and Yi, 2014; Schniering et al., 2019) and is also a “door” for the access of foreign particles, bacteria, viruses into the cell; we believe that the role of thyroid hormone in the immune defense against pathogens or foreign material has become more clear in the last years than it was before, although the mechanisms are not yet known. On the other hand, thyroid hormones are able, among the variety of effects, to modulate the immune function, and a crosstalk exists between the thyroid hormones and the immune system (De Vito et al., 2011; De Vito et al., 2012), and THP-1 monocytes express a high amount of integrin αvβ3 (Dellacasagrande et al., 2000; De Vito et al., 2012).

All these pieces of evidence prompted us to study the capability of thyroid hormones to modulate in THP-1 monocytes from leukemic patients responses typical of immune cells, such as cell migration and proliferation and the possible role of integrin αvβ3 (Cohen et al., 2014). To this aim, we used two different known modulators of cell migration, monocyte chemoattractant protein-1 (MCP-1) and insulin-like growth factor-1 (IGF-1), a growth factor, but also a chemokine produced by injured skeletal muscle (Pillon et al., 2013).

Our data show that thyroid hormones in the presence of either MCP-1 or IGF-1 are able to inhibit cell migration in THP-1 monocytes; the effect is mediated by integrin αvβ3, but with different pathways.

## Materials and Methods

Roswell Park Memorial Institute medium (RPMI-1640), sodium pyruvate (100 mM), L-glutamine (200 mM), streptomycin (100 mg/ml), penicillin (100 U/ml), phosphate buffered saline (PBS; 10 mM Na_2_HPO_4_, 137 mM NaCl, 2.7 mM KCl dissolved in 500 ml of distilled water, pH 7.4), D-glucose (5 mM), *O*-(4-hydroxy-3-iodophenyl)-3,5-diiodo-L-tyrosine sodium salt [3′,5-triiodo-L-thyronine (T_3_)], 3-[4-(4-hydroxy-3,5-diiodophenoxy)-3,5-diiodophenyl]-L-alanine sodium salt [L-thyroxine (T_4_)], tetraiodothyroacetic acid (tetrac), human recombinant IGF-1, Arg-Gly-Asp (RGD) peptide, lipopolysaccharide (LPS; from *Escherichia coli*), *N*_ω_ -nitro-L-arginine methyl ester hydrochloride (L-NAME), nitrosoglutathione (GSNO), dimethyl sulfoxide (DMSO), diphenyleneiodonium chloride, and baicalein (5,6,7-trihydroxyflavone) were supplied by Sigma-Aldrich (St. Louis, MO, United States). Sterile plasticware for cell culture was purchased from Falcon (3V Chimica S.r.l., Roma, Italy); fetal bovine serum (FBS) was obtained from GIBCO (NY, United States). MCP-1 was purchased from PeproTech (NJ, United States). Sterile PBS, D-PBS (Dulbecco’s phosphate buffered saline without calcium and magnesium), was obtained from EuroClone (Italy). PD98059 [a selective inhibitor of MAP kinase kinases (MAPKK), MEK1 and MEK2], wortmannin (a selective irreversible inhibitor of PI3K), and 4,5-diaminofluorescein diacetate (DAF-2DA) were purchased from Alexis Biochemicals (Laufelfingen, Switzerland). Mouse anti-αvβ3 integrin monoclonal antibody (clone LM609) was obtained from Immunological Sciences/Societa’ Italiana Chimici (Rome, Italy).

### Cells in Culture

Human leukemic monocytes THP-1 (ATCC TIB 202) from American Type Culture Collection (Rockville, MD, United States) were grown in a suspension containing RPMI-1640 medium with 10% FBS, 100 μg/ml streptomycin, and 100 U/ml penicillin, in a humidified atmosphere of 5% CO_2_ at 37°C (Pedersen et al., 2007; Lombardo et al., 2013). These cells show a large, round, single-cell morphology. The THP-1 monocytes were passaged twice a week by 1:4 dilutions and reseeded; only cells from passages no. 7–23 were used for the experiments.

### Migration Studies

Migration experiments were carried out by the use of Transwell (Corning) with an 8-μm polycarbonate membrane, 6.5-mm insert 24-well plate with serum-free RPMI-1640 medium containing 0.2% bovine serum albumin (BSA) in both chambers, for 4 h at 37°C (De Vito et al., 2012). THP-1 cells (about 200,000 cells/well) were placed in the upper chamber with RGD (10 μM), tetrac (10 μM), Ab-αvβ3 (8 μg/ml), wortmannin (100 nM), and PD98059 (10 μM), while T_3_ (10^–^^7^–10^–^^9^ M), T_4_ (10^–^^7^–10^–^^11^ M), MCP-1 (100 ng/ml), and IGF-1 (10 nM) were added to the bottom chamber. All αvβ3 inhibitors and L-NAME (1 mM) and GSNO (0.5 mM) were preincubated for 20 min at 37°C. After 4 h of incubation, at 37°C, cells migrated (from the top to the bottom part of the chamber) were counted with a modified Neubauer chamber. At the beginning, migration experiments were carried out with RPMI as medium and in the presence of serum (0.2% FBS) (24). Afterward, since data from literature (Tsaur et al., 2012) show that serum can act as a chemoattractant, we decided to perform experiments in a serum-free medium.

### Proliferation Assay

Cells were seeded in 60 mm × 15 mm Petri dishes with RPMI-1640 and stimulated with RGD, T_4_, and IGF-1 the day after the seeding. Cells were counted after 72 h. The role of integrin αvβ3 on the proliferation of THP-1 cells was studied using RGD peptide (10 μM) as the integrin αvβ3 inhibitor. RGD peptide was preincubated 20 min before adding T_4_ (100 nM), IGF-1 (10 nM), and MCP-1 (100 ng/ml). Moreover, we studied PI3K and MAPK pathways by the use of wortmannin (100 nM) and PD98059 (10 μM) preincubated 20 min before adding T_4_, IGF-1, and MCP-1. Cells were counted with an optical microscope with a Neubauer chamber (Incerpi et al., 2014).

### Nitric Oxide Detection

The production of nitric oxide (NO) in THP-1 cells was measured using DAF-2DA probe. DAF-2DA is a sensitive fluorescent indicator for the detection and bioimaging of NO. It is a cell-permeable derivative of DAF-2. Upon its entry into a cell, DAF-2DA is transformed into the less cell-permeable derivate, DAF-2, by cellular esterases, thus it prevents loss of the signal due to diffusion of the molecule from the cell. In the presence of oxygen, DAF-2 reacts with NO to yield the highly fluorescent triazolofluorescein (DAF-2T). At the time of the experiment, the cell suspension (8 × 10^6^ cells) was washed (1,200 rpm, 10 min) three times with 8 ml of PBS containing 5.0 mM glucose (90 mg/100 ml) to remove the serum, which may affect the action of the fluorescent probe. The incubation with the probe DAF-2DA at the final concentration of 5 μM was carried out for 30 min in the dark at 37°C (López-Figueroa et al., 2000). At the end of the incubation, cells were washed three times, centrifuged at 1,200 rpm for 5 min, and the final cell pellets were resuspended in 4 ml of PBS-glucose. Before the experiments, cells were recovered at 37°C for 30 min in the dark. Some samples of cells were preincubated with L-NAME (1 mM) for 30 min at 37°C. After the recovery, the cells were seeded in a 96-multiwell (200 μl/well) and stimulated with T_4_ (100 nM), MCP-1 (100 ng/ml), ionomycin (2 μM), and LPS (1 mg/ml). Intracellular fluorescence was measured with spectrofluorometer at 37°C (Jasco, Analytical Instruments). Excitation and emission wavelengths were set at 495 and 515 nm, respectively, using 5- and 10-nm slits for the light paths. The measurements were performed at 0, 1, 2, 3, and 4 h.

### The Griess Assay

The measurement of nitrite production was carried out by the Griess assay, a common method for the indirect determination of NO by the spectrophotometric measurement of nitrites. This method requires that nitrates are firstly reduced to nitrite and then determined by the Griess reaction (Ridnour et al., 2000; Colasanti et al., 2004). Herein, cells were seeded in a 24-multiwell and treated with LPS (1 μ g/ml), ionomycin (2 μM), MCP-1 (100 ng/ml), thyroxine (100 nM), and **l**-NAME (1 mM). Nitrite concentrations were tested on THP-1 cells at 4, 24, and 48 h, as described. At different time points, cell suspensions were removed and washed, while supernatants were frozen. In a 96-multiwell plate, a known volume of premixed Griess reagent (1% sulfanilamide, 0.1% naphthylethylenediamine dihydrochloride, and 2.5% H_3_PO_4_) was added to 70 μ of the supernatant of each sample. The reaction was carried out for 15 min at room temperature in the dark. At the end of the reaction, absorbance was measured at 550 nm using ELISA reader (Packard Fusion Microplate Reader). The concentration of nitrite in the supernatants was extrapolated using the calibration curve based on the known concentrations of sodium nitrite (NaNO_2_ 0–50 μ M) reacted with the Griess reagent.

### Western Blot Analysis

THP-1 cells were washed twice with phosphate-buffered saline (PBS), pH 7.4, and lysed in 50 mM Tris, pH 7.4, 150 mM NaCl, 1% Triton-X 100, 0.25% sodium deoxycholate, 1 mM ethylenediaminetetraacetic acid (EDTA), 1 mM ethylene glycol tetraacetic acid (EGTA), 0.5% non-ionic detergent IGEPAL CA-630 (Sigma-Aldrich), 1 mM sodium orthovanadate, 20 mM sodium fluoride, 1 μg/ml leupeptin, 1 mM phenylmethylsulfonyl fluoride, 1 μg/ml pepstatin A, for 30 min in ice. Whole-cell lysates were centrifuged at 6,000 *g* for 10 min at 4°C, and the supernatants were frozen at −80°C. The protein concentrations of cell extracts were determined by the Lowry protein assay (Lowry et al., 1951). The aliquots of cell extracts containing 30 μg of total proteins were resolved on 7% sodium dodecyl sulfate-polyacrylamide gel electrophoresis (SDS-PAGE) and transferred by electroblotting them on nitrocellulose membranes (Whatman GmbH, Dassel, Germany) overnight at 35 V with the Bio-Rad *Trans*-Blot apparatus. For the immunoassays, the membranes were blocked in 3% BSA fraction V (Sigma-Aldrich, St. Louis, MO, United States) in Tween 20-TBS (TTBS)/EDTA (10 mM Tris, pH 7.4, 100 mM NaCl, 1 mM EDTA, 0.1% Tween 20) for 1 h at room temperature. To analyze tyrosine phosphorylation of STAT1, immunoblotting was performed using specific antibodies anti-phosphotyrosine 701-STAT1 diluted in 1% BSA/TTBS-EDTA and then incubated overnight at 4°C. Expression levels of tyrosine phosphorylation of STAT1 were evaluated using corresponding specific antibodies anti-STAT1 or IgG anti-actin as the internal loading control. Antibodies used in the different immunoblottings diluted in 1% BSA/TTBS-EDTA were as follows: rabbit polyclonal antibodies anti-phospho-STAT1 (Tyr701), mouse monoclonal antibodies anti-STAT1, anti-actin rabbit polyclonal antibody. Immune complexes were detected with horseradish peroxidase-conjugated goat anti-rabbit or goat anti-mouse antiserum followed by enhanced chemiluminescence reaction (ECL LiteAblot PLUS, Euro Clone SpA) (Table 1). To reprobe membranes with antibodies having different specificities, nitrocellulose membranes were stripped for 5 min at room temperature with restoring Western blot stripping buffer (Pierce, Rockford, IL, United States) and then extensively washed with TTBS/EDTA.

**TABLE 1:** Antibodies used for Western Blot analysis.

Antibody	Source	Company (catalog no.)
Anti-phospho-STAT1 (Tyr701)	Rabbit polyclonal IgG	Cell Signaling Technology (cat. 9171)
Anti-STAT1	Mouse mAb	Transduction Laboratories (cat. G169 20)
Anti-actin	Rabbit polyclonal ACTA1 antibody	Sigma-Aldrich (cat. A2066)
Anti-rabbit-IgG-HRP conjugate	Goat IgG	Merck Millipore Corporation (cat. AP307P)
Anti-mouse-IgG-HRP conjugate	Goat IgG	Merck Millipore Corporation (cat. AP308P)

### Molecular Docking

In order to analyze the mechanism of interaction between T_4_ and αvβ3 integrin, *in silico* molecular docking simulations have been carried out using the software AutoDock Vina (Trott and Olson, 2010; Di Muzio et al., 2017). This bioinformatics technique is used to predict the way by which two molecules, usually a macromolecule and a small molecule ligand, bind to each other in relation to their chemical structures. We have focused our attention on the interaction between T_4_ and the αvβ3 integrin in its inactive form, both in the presence and in the absence of the peptide RGD, using the three-dimensional structure deposited in the Protein Data Bank with the code 1L5G (Xiong et al., 2002).

### Statistical Analysis

The results reported as means ± SD were analyzed with Student’s *t-*test and with one-way analysis of variance (ANOVA) followed by *post hoc* Bonferroni’s multiple comparison test. Differences were considered significant at *p* < 0.05.

## Results

### Effect of T_4_ on the Migration Induced by MCP-1 and IGF-1 in THP-1 Monocytes

**FIGURE 1:**
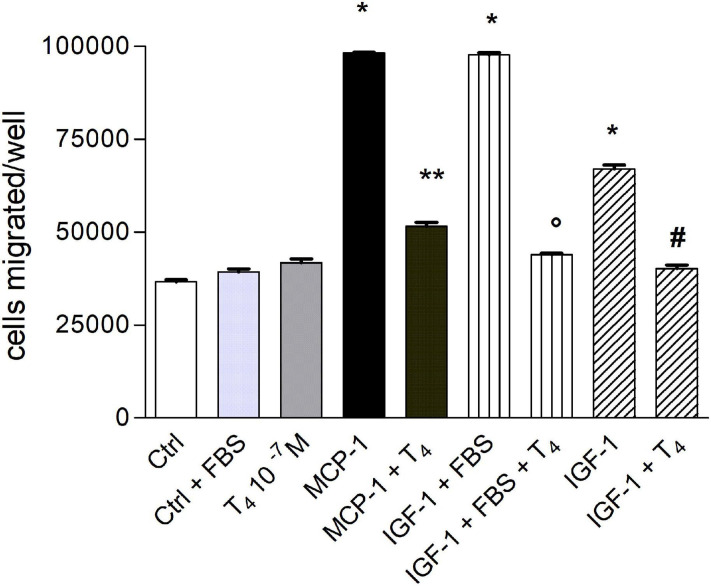
Effect of T_4_ (10^–^^7^ M) on the migration induced by monocyte chemoattractant protein 1 (MCP-1; 100 ng/ml) and insulin-like growth factor-1 (IGF-1; 10^–^^8^ M) with or without fetal bovine serum (FBS) 0.2%. Results are reported as mean ± SD of *n* = 3 different experiments carried out in duplicate. *p < 0.0001 vs. all others; **p < 0.001 vs. MCP-1; °*p* < 0.0001 vs. IGF-1 (0.2% FBS); ^#^*p* < 0.0001 vs. IGF-1.

First of all, we evaluated the effect of T_4_ on THP-1 cell migration in the presence of both MCP-1 and IGF-1. At the beginning, migration experiments were carried out using RPMI as medium and in the presence of serum (0.2% FBS). Afterward, since serum can act as a chemoattractant, we decided to carry out experiments in a serum-free medium. T_4_ alone did not affect cell migration stimulated by either MCP-1 or IGF-1; the stimulatory effect of MCP-1 was higher in comparison to that of IGF-1, according to the key role of MCP-1 as a chemokine (Figure 1). T_4_ inhibited cell migration activated by either MCP-1 or IGF-1 by about 50%.

**FIGURE 2:**
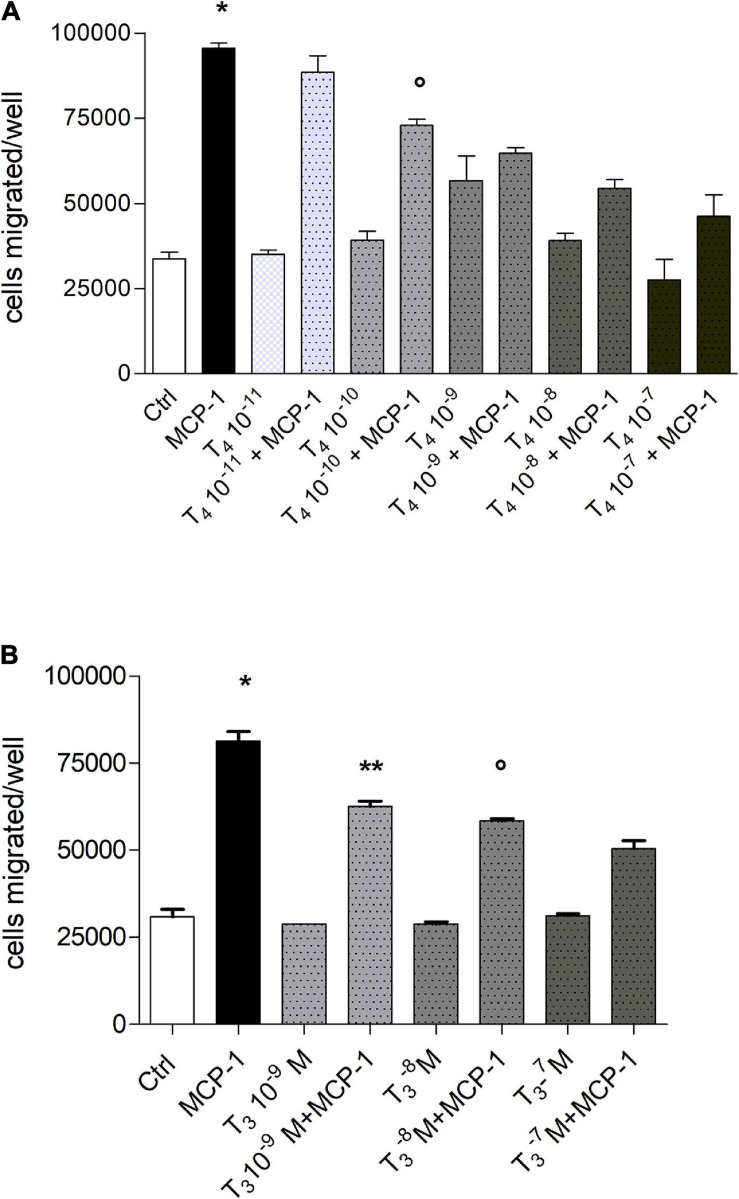
**(A)** Dose–response in a wide concentration range of T_4_ (10^–^^7^–10^–^^11^ M) on the migration induced by monocyte chemoattractant protein 1 (MCP-1; 100 ng/ml). Results are reported as mean ± SD of *n* = 3 different experiments carried out in duplicate. *p < 0.01, at least, vs. all but T4 10^–^^11^ M + MCP-1; °*p* < 0.01 vs. MCP-1 and T_4_ 10^–^^8^ M + MCP-1. **(B)** Dose–response of T_3_ (10^–^^7^–10^–^^9^ M) in the migration induced by MCP-1 (100 ng/ml). Results are reported as mean ± SD of *n* = 3 different experiments carried out in duplicate. *p < 0.001vs. all; **p < 0.001 vs. MCP-1; °*p* < 0.05 vs. T_3_ 10^–^^7^ M + MCP-1.

Experiments of dose–response of T_4_ (10^–^^11^–10^–^^7^ M) show that the inhibitory effect of the hormone on the migration stimulated by MCP-1 was dose-dependent: T_4_ (10^–^^7^ M) prevented cell migration induced by either MCP-1 or IGF-1. The inhibitory effect of the thyroid hormone was significant starting at 10^–^^10^ M (*p* < 0.01 with respect to MCP-1; Figure 2A). All experiments to be shown in the following were carried out with T_4_ 10^–^^7^ M, the physiological concentration of T_4_. We also studied the effect of T_3_ on cell migration induced by MCP-1 (Figure 2B). Our data show that T_3_ alone, at different concentrations (10^–^^7^–10^–^^9^ M) did not affect cell migration, but when given together with MCP-1, T_3_, as well as T_4_, was able to prevent cell migration in a dose-dependent way starting at 10^–^^9^ M (*p* < 0.001 with respect to MCP-1), but its inhibitory effect was lower with respect to that of T_4_, although significant at all concentrations tested (Figure 2B); this may be due to the lower affinity of T_3_ for the integrin with respect to T_4_.

### Mechanism of Inhibition by Thyroid Hormones of Cell Migration Induced by IGF-1 or MCP-1 in THP-1 Monocytes: Role of Integrin αvβ3

**FIGURE 3:**
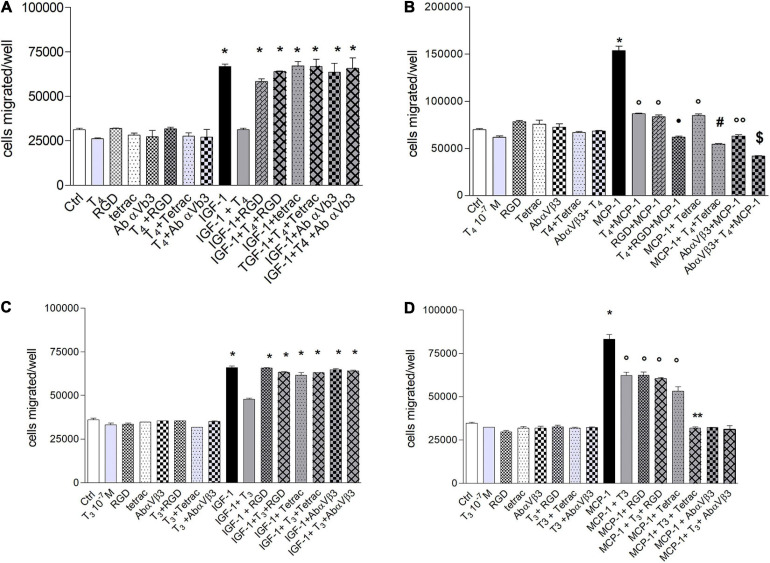
**(A)** Effect of T_4_ (100 nM), RGD, tetrac, and Ab αvβ3 on the migration of THP-1 cells, induced by insulin-like growth factor-1 (IGF-1). RGD (10 μM), tetrac (10 μM), and Ab-αvβ3 (8 μg/ml) were preincubated 20 min before adding T_4_ (100 nM) and IGF-1 (10^–^^8^ M). Results are reported as mean ± SD of *n* = 3 different experiments carried out in duplicate. *p < 0.001 vs. all. **(B)** Effect of T_4_ (100 nM), RGD, tetrac, and Ab-αvβ3 on the migration of THP-1 cells, induced by monocyte chemoattractant protein 1 (MCP-1). RGD (10 μM), tetrac (10 μM), and Ab-αvβ3 (8 μg/ml) were preincubated 20 min before adding T_4_ (100 nM) and MCP-1 (100 ng/ml). Results are reported as mean ± SD of *n* = 4 different experiments carried out in duplicate. *p < 0.001 vs. all; °*p* < 0.05 vs. Ctrl; ^*^p < 0.001 vs. MCP-1 + RGD; ^#^*p* < 0.001 vs. MCP-1 + tetrac; °°*p* < 0.05 vs. MCP-1 + tetrac; ^§^ *p* < 0.01 vs. MCP-1 + Ab-αvβ3. **(C)** Effect of T_3_ (100 nM), RGD, tetrac, and Ab-αvβ3 on the migration of THP-1 cells, induced by IGF-1. RGD (10 μM), tetrac (10 μM), and Ab-αvβ3 (8 μg/ml) were preincubated 20 min before adding T_3_ (100 nM) and IGF-1 (10^–^^8^ M). Results are reported as mean ± SD of *n* = 2 different experiments carried out in duplicate. *p < 0.001 vs. all. **(D)** Effect of T_3_ (100 nM), RGD, tetrac, and Ab-αvβ3 on the migration of THP-1 cells, induced by MCP-1. RGD (10 μM), tetrac (10 μM), and Ab-αvβ3 (8 μg/ml) were preincubated 20 min before adding T_3_ (100 nM) and MCP-1 (100 ng/ml). Results are reported as mean ± SD of *n* = 4 different experiments carried out in duplicate. *p < 0.001 vs. all; °*p* < 0.05 vs. Ctrl; **p < 0.001 vs. MCP-1 + tetrac.

The αvβ3 integrin is involved in the invasion and migration of different cells (Cohen et al., 2014; Liu et al., 2016), besides being the plasma membrane receptor for thyroid hormone (Bergh et al., 2005) and a co-receptor for IGF-1 (Clemmons et al., 2007). Therefore, we also tested the effect of thyroid hormone on the migration induced by IGF-1 (10 nM). The possible role of integrin αvβ3 was studied using three well-known integrin αvβ3 inhibitors: Arg-Gly-Asp peptide (RGD), tetrac, a metabolite of thyroid hormone and a probe for the integrin αvβ3 and a human monoclonal antibody-αvβ3, LM-609. In the presence of IGF-1, the inhibitors of integrin αvβ3 were able to prevent the inhibitory effect of the hormone on the IGF-1-induced migration (Figure 3A). At variance with this, in the presence of MCP-1, T_4_ and the inhibitors of the integrin, RGD, tetrac, or antibody-αvβ3, gave rise to a potentiation of the inhibitory effect of the migration stimulated by MCP-1 (Figure 3B).

We carried out experiments with T_3_, to verify whether there were differences in the behavior of the two hormones, since T_3_ binds integrin with about two orders of magnitude in the affinity lower with respect to T_4_ (Bergh et al., 2005). The results obtained in the presence of T_3_, in the cell migration using IGF-1 as a chemoattractant, are similar to those of T_4_ in the presence of IGF-1, and all of the αvβ3 integrin inhibitors prevented the effect of T_3_, analogously to T_4_ (Figure 3C).

T_3_ inhibited the migration induced by MCP-1, with a lower effect in comparison to T_4_ (Figure 3D) and a different behavior with respect to T_4_ (Figure 3B). In fact, all αvβ3 inhibitors were able to prevent the migration induced by MCP1, but T_3_ + MCP-1 behaved in a different way in the presence of either RGD or tetrac or the antibody. In particular, two levels of inhibition of cell migration appear to be present in this type of experiments. In fact, the first level of inhibition, although significant with respect to MCP-1 alone, was found for MCP-1 in the presence of T_3_, RGD, T_3_ + RGD, or tetrac, although with a trend for the last to a higher effect. The second level of inhibition of migration was more effective and brought back the migration to basal level when MCP-1 was in the presence of T_3_ and either tetrac or antibody or T_3_ plus antibody (Figure 3D).

### Signal Transduction of Migration Stimulated by Insulin-Like Growth Factor-1 and Monocyte Chemoattractant Protein-1 in THP-1 Monocytes in the Presence of Thyroid Hormones

**FIGURE 4:**
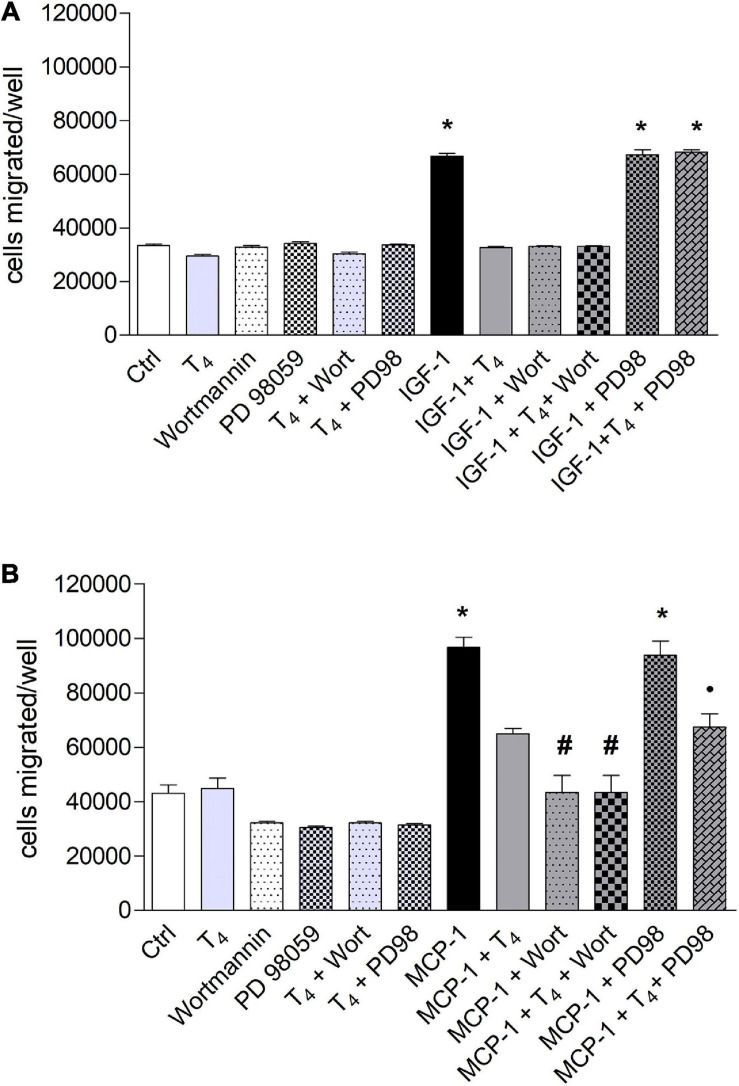
Effect of T_4_ on the migration of THP-1 cells induced by either insulin-like growth factor-1 (IGF-1) **(A)** or monocyte chemoattractant protein 1 (MCP-1) **(B)**. Possible role of mitogen-activated protein kinase (MAPK) and phosphoinositide 3-kinase (PI3K) pathways. **(A)** PD98059 (10 μM) and wortmannin (100 nM) were preincubated 20 min before the addition of T_4_ (10^–^^7^ M) and IGF-1 (10^–^^8^ M). Results are reported as mean ± SD of *n* = 3 different experiments carried out in duplicate. *p < 0.001 vs. all others. **(B)** PD98059 (10 μM) and wortmannin (100 nM) were preincubated 20 min before adding T_4_ (10^–^^7^ M) and MCP-1 (100 ng/ml). Results are reported as mean ± SD of *n* = 4 different experiments carried out in duplicate. *p < 0.001 vs. all the others; ^#^*p* < 0.05 vs. T_4_ + MCP-1; ^*^p < 0.05 vs. PD98 + MCP-1.

**FIGURE 5:**
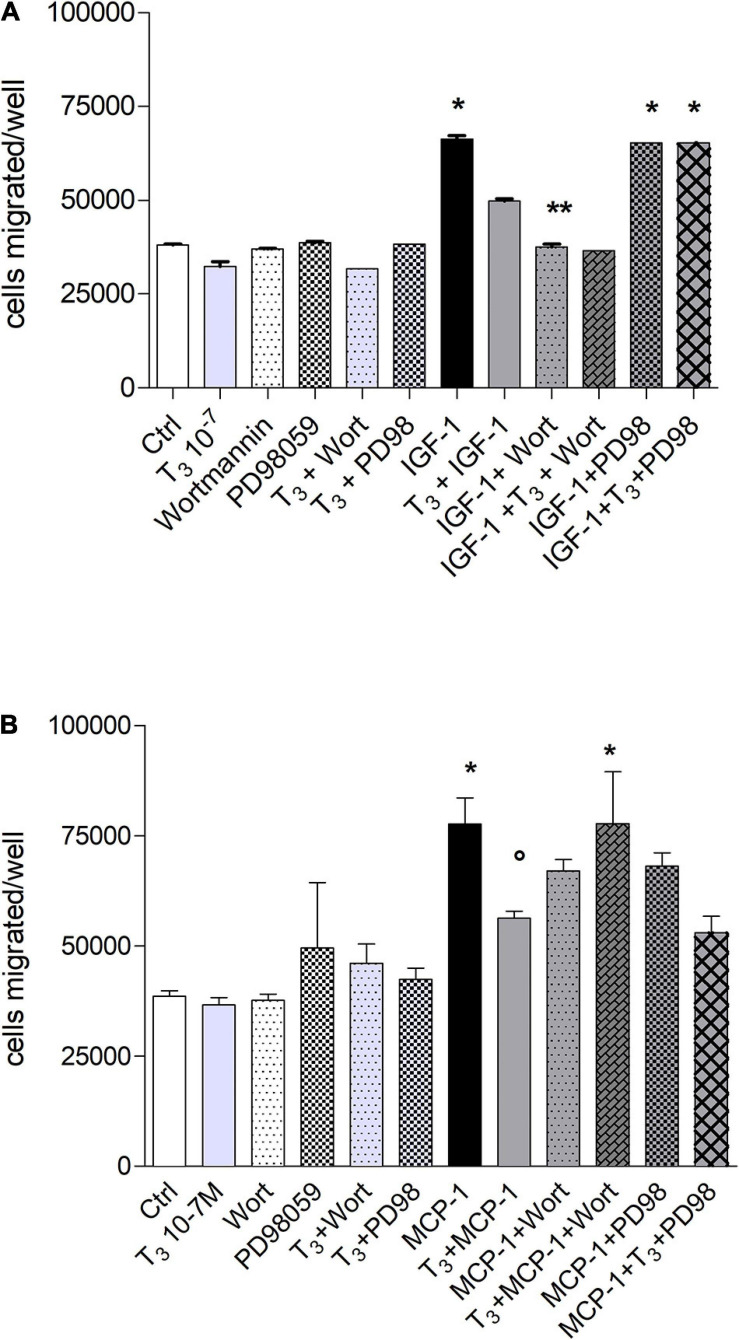
Effect of T_3_ (10^−7^ M) on the migration of THP-1 cells induced by either insulin-like growth factor-1 (IGF-1) **(A)** or monocyte chemoattractant protein 1 (MCP-1) **(B)**. PD98059 (10 μM) and wortmannin (100 nM) were preincubated 20 min before adding T_3_ (10^–^^7^ M) and MCP-1 (100 ng/ml). Results are reported as mean ± SD of *n* = 2–4 different experiments carried out in duplicate. **(A)** *p < 0.05, at least, vs. the others; **p < 0.001 vs. T_3_ + IGF-1; **(B)** *p < 0.05 at least, vs. all the others but MCP-1 + PD98059; °*p* < 0.05 vs. MCP-1 + T_3_ + Wort and vs. MCP-1.

The signal transduction of both T_4_ and MCP-1 shows the involvement of PI3K pathway for MCP-1 and MAPK pathway downstream the interaction of T_4_ with integrin αvβ3 (Cohen et al., 2014; Lee et al., 2015; Shinderman-Maman et al., 2016). For this reason, we focused our study on these two pathways, by a pharmacological approach, using MAPK and PI3K inhibitors, PD98059 and wortmannin, respectively. IGF-1 stimulated cell migration through PI3K pathway. Interestingly, in the presence of the MAPK inhibitor, PD98059, T_4_ did not inhibit the cell migration induced by IGF-1 (Figure 4A). These results indicate that T_4_ prevents cell migration stimulated by IGF-1 through the MAPK pathway. MCP-1 induced cell migration through the PI3K pathway, since in the presence of wortmannin, the MCP-1-induced cell migration was inhibited up to the basal level (Figure 4B). As to the inhibitory effect of the thyroid hormone, T_3_, the PI3K pathway is also involved. In fact, wortmannin prevented the inhibitory effect of T_3_ on the cell migration stimulated by MCP-1 (Figure 5B). T_3_ and T_4_ behave in a similar way as to the role of the MAPK pathway in the inhibitory effect on migration induced by IGF-1 (Figures 4A, 5A). The results obtained from the study of the signal transduction indicate that both MCP-1 and IGF-1 induced cell migration through the PI3K pathway, but T_3_ and T_4_ acted in a different way: T_4_ prevented cell migration induced by IGF-1 through MAPK, while this pathway is not involved in the inhibitory effect of T_3_ in cell migration induced by MCP-1, and the presence of PD98059 did not significantly affect the inhibition of T_3_ on MCP-1-stimulated cell migration. Wortmannin, instead, did prevent the inhibitory effect of T_3_ on the migration induced by MCP-1, in agreement with the reported binding of T_3_ to the S1 site of the integrin αvβ3 and downstream activation of the PI3K pathway (*p* < 0.05; Figure 5B; Lin et al., 2009).

### Thyroid Hormones Modulate THP-1 Monocyte Proliferation Induced by IGF-1 and MCP-1: Role of Integrin αvβ3

**FIGURE 6:**
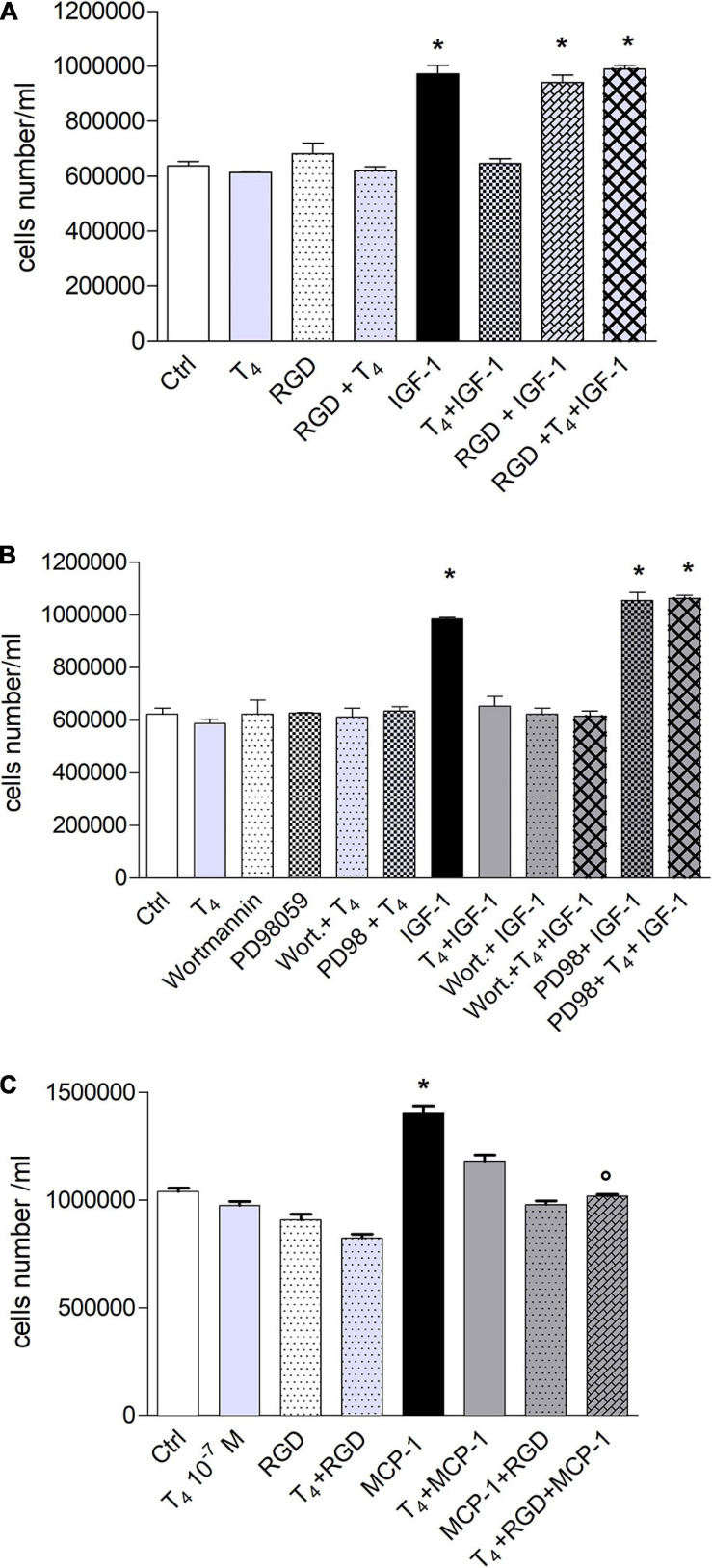
Effect of T_4_ on the proliferation of THP-1. **(A)** Effect of RGD on the proliferation of THP-1 cells, induced by insulin-like growth factor-1 (IGF-1) and IGF-1 + T_4_. RGD (10 μM) was preincubated 20 min before T4 (10^–^^7^ M) and IGF-1 (10^–^^8^ M). Cells were counted 72 h after the stimulation. Results are reported as mean ± SD of *n* = 2 different experiments carried out in duplicate. *p < 0.001 vs. all others. **(B)** Role of mitogen-activated protein kinase (MAPK) and phosphoinositide 3-kinase (PI3K) pathways in THP-1 cell proliferation, induced by IGF-1 and IGF-1 + T_4_. PD98059 (10 μM) and wortmannin (100 nM) were preincubated 20 min before adding T4 (10^–^^7^ M) and IGF-1 (10^–^^8^ M). Results are reported as mean ± SD of *n* = 3 different experiments carried out in duplicate. *p < 0.001 vs. all. **(C)** Effect of T_4_, monocyte chemoattractant protein 1 (MCP-1), MCP-1 + T_4_ on the proliferation of THP-1 monocytes. RGD (10 μM) was given to the cells 20 min before the hormone and MCP-1 and counted after 72 h from seeding at confluency. *p < 0.001 vs. all, °*p* < 0.001vs. T_4_ + MCP-1.

IGF-1 enhances cell proliferation and survival (Laron, 2001; Hakuno and Takahashi, 2018), and IGF-1 significantly increased the proliferation of THP-1 monocytes with respect to control, and T_4_ inhibited it; this effect was due to αvβ3 integrin (Figure 6A). In particular, we evaluated the role of integrin αvβ3 in cell proliferation; RGD alone did not affect cell proliferation but was able to remove the inhibitory effect of thyroid hormone (Figure 6A). These results are in agreement with data obtained from cell migration experiments, since both hormone responses, cell migration and proliferation, are mediated by integrin αvβ3. After evaluating the inhibitory effect of T_4_ and the role of integrin αvβ3 in cell proliferation, we studied whether MAPK and PI3K pathways might be involved in this process by a pharmacological approach. We previously published that IGF-1 stimulated L6 myoblast proliferation through PI3K pathway, while the thyroid hormone prevented this process through the MAPK pathway (Incerpi et al., 2014). Moreover, as shown before (Figure 6B), these two pathways are involved in THP-1 proliferation induced by IGF-1. The results obtained are similar to those of migration experiments: IGF-1 stimulated cell proliferation through PI3K, as already reported in the literature (Baserga et al., 1997; Riedemann and Macaulay, 2006; Incerpi et al., 2014), whereas the inhibitory effect of T_4_ in cell proliferation was mediated by MAPK, since PD98059, in the presence of IGF-1 and T_4_, was able to revert the inhibitory effect of the thyroid hormone (Figure 6B).

MCP-1 also stimulated THP-1 cell proliferation, and the effect was inhibited by RGD, indicating that also, in this case, we have a genomic response mediated by the plasma membrane integrin αvβ3. Again, RGD was unable to bring back the inhibition of proliferation induced by T_4_ but eventually increased it, a response parallel to the results on cell migration carried out with this chemokine (Figure 6C).

### Role of Reactive Oxygen and Nitrogen Species in the Migration Induced by Monocyte Chemoattractant Protein-1 in THP-1 Monocytes

**FIGURE 7:**
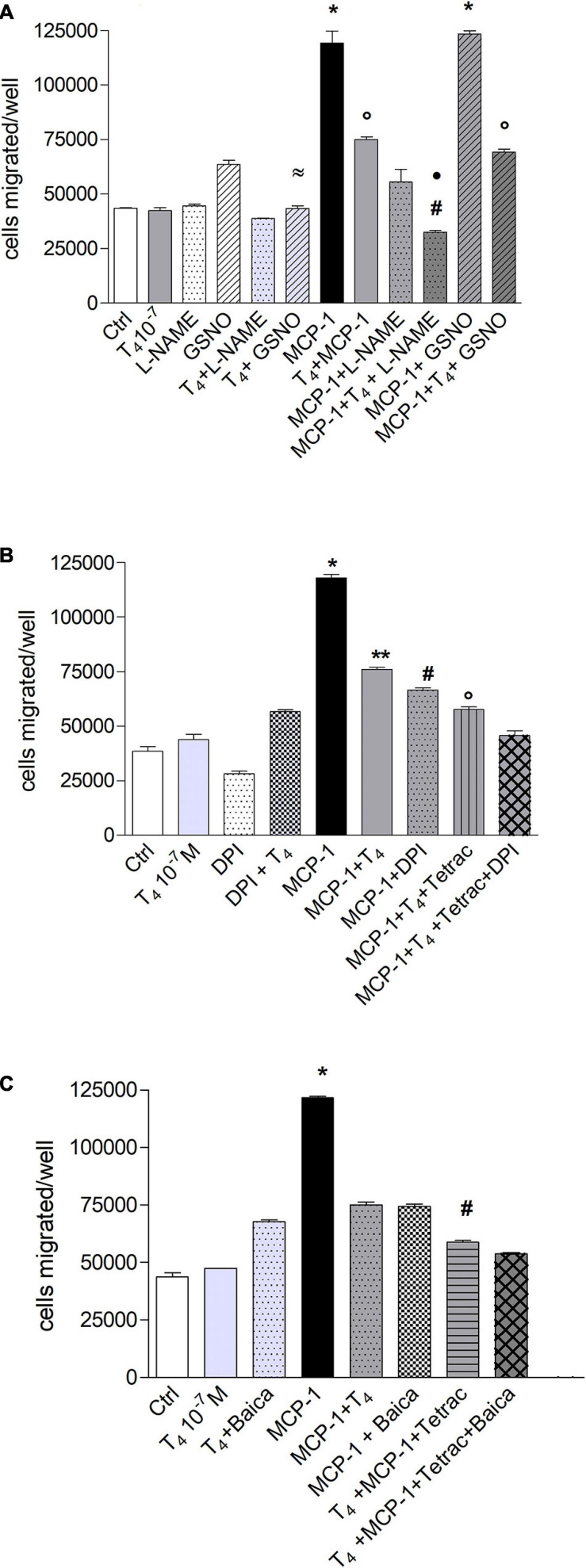
Possible effects of reactive oxygen species (ROS) and reactive nitrogen species (RNS) in the signal transduction of thyroid hormone inhibition of monocyte chemoattractant protein 1 (MCP-1)-mediated migration in THP-1 monocytes. **(A)** Effect of *N*_ω_ -nitro-L-arginine methyl ester hydrochloride (L-NAME; 1 mM) and nitrosoglutathione (GSNO, 0.5 mM) on the migration of THP-1 monocytes stimulated by MCP-1 and modulated by thyroid hormone (T_4_). The inhibitor of nitric oxide (NO), L-NAME, and the NO donor, GSNO (0.5 mM), were preincubated 20 min at 37°C before the addition of T_4_ (10^–^^7^ M) and MCP-1 (100 ng/ml). Results are reported as mean ± SD of *n* = 2 different experiments carried out in duplicate. *p < 0.001 vs. all; °*p* < 0.05, at least, vs. MCP-1 + L-NAME; ^#^*p* < 0.001 vs. T4 + MCP-1; ^*^p < 0.001 vs. MCP-1 + L-NAME; ^≈^*p* < 0.001 vs. GSNO. **(B)** Effect of diphenylene iodonium (DPI; 20 μM) on the inhibition of migration of THP-1 cells by thyroid hormone on MCP-1. All inhibitors were preincubated 20 min before addition of T_4_ (10^–^^7^ M) and MCP-1 (100 ng/ml). Results are reported as mean ± SD of *n* = 3 different experiments carried out in duplicate. *p < 0.001 vs. all, **p < 0.01, at least, vs. MCP-1 + DPI, MCP-1 + T4 + tetrac, MCP-1 + T4 + tetrac + DPI, ^#^p < 0.001 vs. T4 + MCP-1 + tetrac and T4 + MCP-1 + tetrac + DPI, °*p* < 0.001 vs. T4 + MCP-1 + tetrac + DPI. **(C)** Effect of baicalein (10 μM) on the migration of THP-1 cells stimulated by MCP-1 and in the presence of thyroid hormone. Baicalein was preincubated 20 min before the addition of T_4_ (10^–^^7^ M) and MCP-1 (100 ng/ml). Results are reported as mean ± SD of *n* = 3 different experiments carried out in duplicate. *p < 0.001 vs. all; ^#^*p* < 0.001 vs. MCP-1 + Baica, MCP-1 + T_4_, T4 + MCP-1 + tetrac + Baica.

Migration of monocytes is required for routine immunological surveillance of tissues and their entry into inflamed sites, and NO plays a key role during inflammation (Sharma et al., 2007). Therefore, we studied the possible involvement of NO in the migration induced by MCP-1, as already reported (Biswas et al., 2001), and we tried to understand whether the effect of thyroid hormones might be related to a modulation of nitrite or reactive oxygen species (ROS) production. To this aim, we carried out experiments of cell migration using the NO inhibitor, L-NAME (1 mM), and an NO donor, GSNO (0.5 mM). The results of experiments carried out, with MCP-1 and T_4_, show that L-NAME was able to potentiate the inhibitory effect of T_4_ in the presence of MCP-1. As to the NO donor, GSNO alone stimulated cell migration with respect to control (*p* < 0.01), but GSNO in the presence of T_4_ was able to bring the migration back to the basal level. GSNO did not affect the migration induced by MCP-1 (Figure 7A).

To assess the possible role of ROS, we used diphenylene iodonium (DPI), a nicotinamide adenine dinucleotide phosphate (NADPH) oxidase inhibitor, and a metabolite of thyroid hormones, tetrac, known to inhibit the interaction of the hormone with the integrin αvβ3 through binding to the RGD site or in its close proximity. DPI inhibits the migration induced by MCP-1, pointing to an involvement of the NADPH oxidase and ROS production in the cell migration stimulated by MCP-1. In the presence of DPI, T_4_, and tetrac, we found a potentiation of the inhibitory effect on cell migration stimulated by MCP-1 (Figure 7B). These results indicate the involvement of ROS and NO in the cell migration activated by MCP-1 and perhaps a crosstalk between NADPH oxidase and integrin αvβ3, as previously hypothesized (Chuang et al., 2004; De Vito et al., 2012; Gnocchi et al., 2012). The high level of metabolic activity of the cells results in an increase of ROS that makes the antioxidant defense an important factor (Lombardo et al., 2013). We also studied the effect of baicalein, a flavonoid from the roots of *Scutellaria baicalensis*, on THP-1 cell migration. Baicalein is a strong antioxidant also at very low concentrations in THP-1 monocytes, reported to be able to modulate also NO production (Seo et al., 2011; Lombardo et al., 2013; Caioli et al., 2016). Baicalein was able to inhibit significantly the cell migration induced by MCP-1, as well as DPI (Figures 7B,C). In addition, the inhibitory effect of baicalein was significantly potentiated in the condition T_4_ + MCP-1 + tetrac, similar to that of DPI (Figures 7B,C).

**FIGURE 8:**
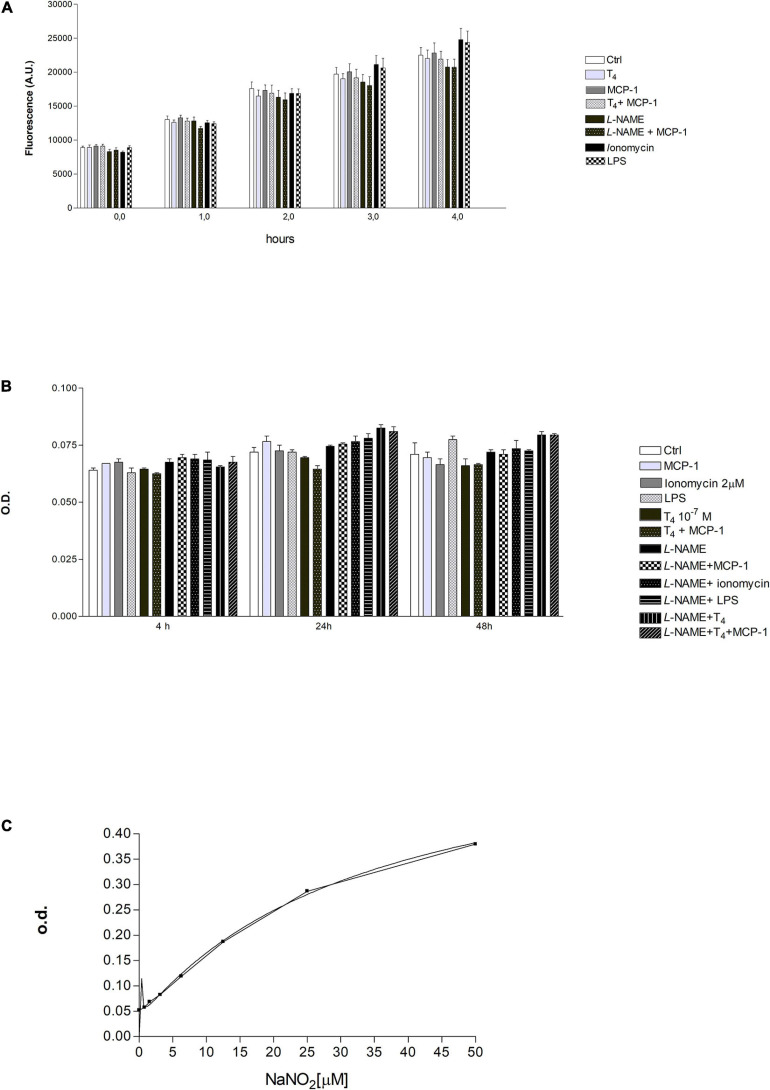
**(A)** Effect of T_4_ (10^–^^7^ M) and monocyte chemoattractant protein 1 (MCP-1) (100 ng/ml) on reactive nitrogen species (RNS) production in THP-1 monocytes. *N*_ω_ -nitro-L-arginine methyl ester hydrochloride (L-NAME; 1 mM), ionomycin (2 μM), lipopolysaccharide (LPS; 1 μg/ml). Results are reported as mean ± SD of *n* = 3 different experiments. None of the differences was significant within the same time frame. **(B)** Measurements of optical density (OD) by ELISA reader, after 4, 24, and 48 h of stimulation with T4 (10^–^^7^ M), MCP-1 (100 ng/ml), L-NAME (1 mM), ionomycin (2 μM), and LPS (0.1 μg/ml). Results are reported as mean ± SD of *n* = 3 different experiments. None of the reported differences was significant. **(C)** Concentration of nitrite in the supernatants of samples of Figure 7B was extrapolated using a calibration curve based on the known concentrations of sodium nitrite (NaNO_2_; 0–50 μM) reacted with the Griess reagent. The graph shows two calibration curves carried out for two different experiments.

We also evaluated the production of NO in THP-1 human leukemic monocytes using the DAF-2DA fluorescent probe. Experiments were carried out, using ionomycin and LPS as positive controls, at 0, 1, 2, and 4 h. There was an increase of reactive nitrogen species (RNS) from 0 to 4 h, but we did not observe a significant difference between samples treated in the same time frame (Figure 8A). After that, the measurement of nitrite production, in THP-1 cells, was carried out by the Griess reaction (Figures 8B,C). This method was used to obtain a quantitative measurement of NO at 4–24 and 48 h after treatment. Our results show that the nitrite concentration was almost the same from 4 to 48 h and was very low (<5 μM). Although the results obtained suggest an involvement of NO in THP-1 cell migration, it was not easy to determine by the use of the fluorescent probe and the Griess assay. Probably, the THP-1 cells produce very little NO or not enough to be detected by the methods used in our laboratory.

### T_4_ Activates Signal Transducer and Activator of Transcription-1α, but It Prevents Its Tyrosine Phosphorylation When Stimulated by Insulin-Like Growth Factor-1

**FIGURE 9:**
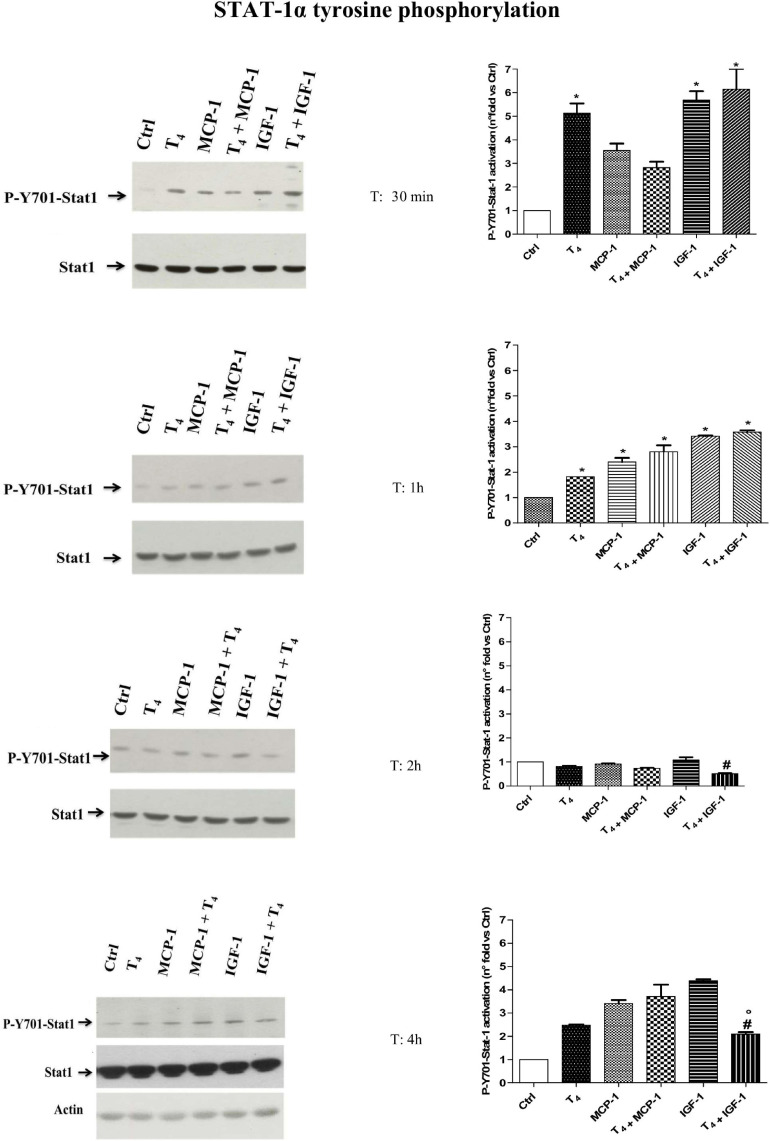
Western blot and densitometric analysis of P-Y701-Stat1 activation after 30 min, 1 h, 2 h, and 4 h of T_4_ (10^–^^7^ M), monocyte chemoattractant protein 1 (MCP-1; 100 ng/ml), and insulin-like growth factor-1 (IGF-1; 10^–^^8^ M) stimulation of THP-1 cells. Western blot of one of two independent experiments was reported. The densitometric analysis is reported as mean ± SD of *n* = 2 different experiments. *p < 0.01 vs. Ctrl; ^#^*p* < 0.05, at least, vs. IGF-1; °*p* < 0.05 vs. T_4_ + MCP-1.

STAT-1α is a signal transducer and activator of transcription that mediates cellular responses to IFNs, cytokines, and other growth factors. T_4_ activates STAT (Lin et al., 1999a, b; Shih et al., 2004; Davis et al., 2005; Staab et al., 2012); therefore, we studied the possible modulation of STAT-1α in THP-1 monocytes after stimulation with T_4_ in the presence and absence of either MCP-1 or IGF-1 through a Western blot analysis at different times: 30 min, 1 h, 2 h, and 4 h, the time of migration experiments. Our results were normalized with respect to the level of expression of STAT1, which was constant for all conditions and times. After 30 min of stimulation, there was a significant increase of STAT-1α tyrosine phosphorylation (P-Y701-STAT1) in all conditions, with respect to control, but after 1 h, there was a decrease that reached the basal value at 2 h to increase again at 4 h, in good agreement with similar values reported for different cells (Lin et al., 1999a; Figure 9). Interestingly, T_4_ was able to prevent STAT-1α tyrosine phosphorylation induced by IGF-1 at 2 h that was still present after 4 h. At this time, we observed a second wave of STAT-1α tyrosine phosphorylation in the other conditions, with respect to 30 min, indicating a more stable activation. P-Y701-STAT-1α is an important transcription factor related to the activation of inducible nitric oxide synthase (iNOS) in macrophages as reported (Lin et al., 1999a, b; Arjcharoen et al., 2007).

### Molecular Docking

**FIGURE 10:**
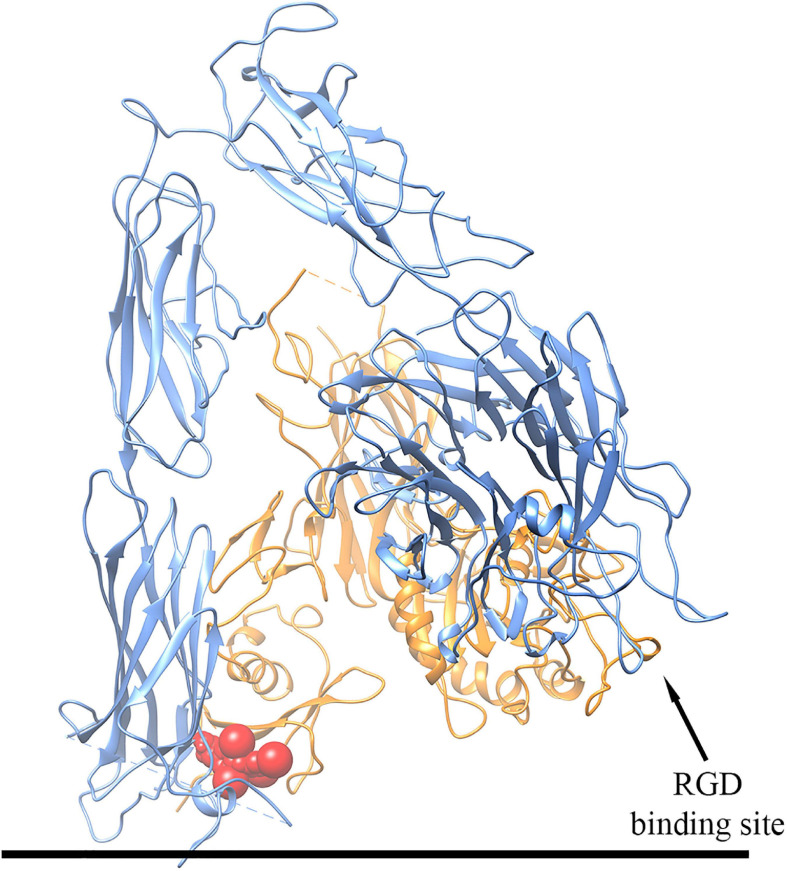
Schematic representation of the three-dimensional structure of the putative complex between T_4_ and the αvβ3 integrin obtained by docking simulations. The αv subunit is colored in blue, the β3 subunit in orange, and T_4_ in red. For details, see text.

In order to analyze the mechanism of interaction between T_4_ and αvβ3 integrin, *in silico* molecular docking simulations have been carried out using the software AutoDock Vina (Trott and Olson, 2010; Di Muzio et al., 2017). We have focused on the interaction between T_4_ and the αvβ3 integrin in its inactive form, both in the presence and in the absence of the peptide RGD, using the three-dimensional structure deposited in the Protein Data Bank with the code 1L5G (Xiong et al., 2002). Our results show that T_4_ mainly binds at the interface between the two αvβ3 integrin subunits in the basal part of the macromolecule next to the cell membrane, a site that is different from the RGD binding site. Interestingly, T_4_ was able to bind to this site both in the presence and in the absence of the RGD peptide. In particular, as mentioned above, the putative T_4_ binding site is located in the extracellular space but very close to the plasma membrane, and interacting with both integrin subunits may stabilize the inactive conformation of the αvβ3 integrin (Figure 10). In fact, during integrin activation, the basal domains of the two integrin subunits must move away from each other (Xiong et al., 2002), a process that would be inhibited by T_4_ binding in this region, at the interface between the two subunits. These results suggest a mechanistic interpretation of the data of migration induced by MCP-1 in the presence of T_4_ and the αvβ3 integrin inhibitor RGD. In fact, experimental data of migration show that both RGD and T_4_ inhibited the migration induced by MCP-1, and there was a significant potentiation in the inhibition of the migration when T_4_ was in the presence of RGD or other inhibitors of the integrin. As mentioned above, this effect could be due to the presence on the integrin of a binding site for the hormone, different from the RGD site. At variance with results obtained with MCP-1, where both T_4_ and RGD inhibit the migration induced by MCP-1 resulting in a potentiation of the inhibitory effect, data of migration in the presence of IGF-1 are different, but since no crystallographic structure is available for the active form of αvβ3 integrin, molecular docking simulations could not be performed on this integrin state. Our data on migration induced by IGF-1 in THP-1 monocytes are in agreement with previous data, published from different laboratories including our own, on the inhibitory effect of T_4_ on the IGF-1-mediated actions in different cells (Incerpi et al., 2014) and papers from other groups, where it is reported that preincubation with RGD, but also tetrac and the Ab-αvβ3, prevented the hormone effect (Jones et al., 1996; Moeller et al., 2006; Moeller and Broecker-Preuss, 2011).

## Discussion

This paper shows for the first time the capability of thyroid hormones to modulate directly or through the integrin αvβ3 a typical response of the immune cells in THP-1 monocytes: cell migration stimulated by chemokines, MCP-1 and IGF-1 (Pillon et al., 2013). We previously reported that thyroid hormones, mainly T_4_, inhibit the glucose uptake and cell proliferation stimulated by IGF-1 in L6 myoblasts from rat skeletal muscle, and the effect is mediated by the integrin αvβ3 (Incerpi et al., 2014). The modulation by thyroid hormone of cell migration mediated by the integrin αvβ3 in THP-1 monocytes was also previously reported (De Vito et al., 2012). We wanted to assess whether this inhibition of thyroid hormones on the effects of IGF-1 might be found also in different cells and for different responses, as reported for other growth factors (Shih et al., 2004). Thyroid hormone, mainly T_4_, is able to inhibit the migration stimulated by a high concentration of MCP-1 and IGF-1, and the effect in both cases is mediated by integrin αvβ3, but with different mechanisms. The migration of THP-1 monocytes is very similar by using either MCP-1 or IGF-1 as chemoattractants, although the crosstalk between integrin αvβ3 and T_4_ is quite different. In fact, when THP-1 monocytes were treated with MCP-1 and T_4_, in the presence of the inhibitors of the binding of T_4_ to the integrin, RGD, tetrac, or the antibody for the integrin αvβ3, the inhibition of migration was not reverted but showed a trend to a potentiation. When IGF-1 was used as a chemoattractant, the inhibitors of the T_4_–integrin αvβ3 interaction, RGD, tetrac, Ab αvβ3 completely reverted the inhibition of the migration induced by T_4_, suggesting that T_4_ in that case binds the integrin at the RGD site or in close proximity to, as expected (Cheng et al., 2010; Davis et al., 2016).

As to the mechanism by which T_4_ inhibits the migration induced by MCP-1, we hypothesize that T_4_ binds a site of the integrin different from the RGD site, so that when RGD, tetrac, or Ab αvβ3 binds to the integrin, the inhibition shows a trend to a potentiation. The integrin–hormone receptor interaction has several interesting features that allow a sort of specialization of the ligand-binding domain so that functions regulated from that domain are distinguished from those of RGD recognition site ligands (Lin et al., 2009). These results are confirmed by the docking experiments.

Within the iodothyronine receptor domain on the integrin αvβ3, there are two hormone binding sites S1 and S2; T_3_ interacts with S1, activating PI3K signaling and Src kinase. Both T_3_ and T_4_ bind S2, leading to ERK1/2 activation of pathways such as proliferation. The effect of T_4_ on cell proliferation is inhibited by both RGD and tetrac, whereas those of T_3_ through S2 on cell proliferation are inhibited only by tetrac. Therefore, at S2, T_4_ appears to be more effective than T_3_ (Lin et al., 2009).

Nanotetrac, a particulate form of tetrac, that binds at the RGD site of integrin αvβ3, inhibits the expression of genes for certain chemokines (such as fractalkine, CX3CL1) and chemokine receptors (such as CX3CR1) that have been identified as targets for the development of anti-inflammatory drugs. Thyroid hormone and tetrac formulations may also have clinically relevant anti-inflammatory effects, but this topic has not been studied for the time being. We can hypothesize that thyroid hormone binds directly to integrin through the fractalkine (FKN) site (Davis et al., 2013).

The unexpected results on the potentiation of the inhibitory effect of the thyroid hormones on MCP-1-induced cell migration lead us to hypothesize a direct interaction between thyroid hormone and this chemokine. A direct binding of thyroid hormone to a chemokine was previously reported. In fact, data from literature indicate an interaction between T_4_ and the macrophage migration inhibitory factor (MIF), a pro-inflammatory cytokine (Al-Abed et al., 2011). The authors investigated the interaction between T_4_ and MIF by molecular modeling, and they identified T_4_ as a potential endogenous ligand for MIF. Integrin αvβ3 was reported to bind directly to fibroblast growth factor (FGF), without the involvement of the RGD site. In addition, the group of Takada and Takada demonstrated that an integrin binding defective-FGF1 mutant (R50E) significantly reduced the capability of the growth factor to cause cell proliferation and migration, and they proposed that the direct binding of integrin to FGF1 is the basis of the crosstalk between integrin and FGF1 (Mori et al., 2008). The same authors have also shown that the chemokine domain of fractalkine (FKN-CD) binds to the RGD site of αvβ3 integrin and gives rise to a ternary complex (integrin-FKN-CX3CR1) important for both the downstream signaling of CX3CR1 and integrin activation (Fujita et al., 2014). A similar ternary complex is formed by IGF-1–IGF1R–integrin αvβ3, critical for the downstream signaling of IGF-1 and integrin activation (Takada et al., 2017). MCP-1 expression is increased by Cyr61, an angiogenic factor. The effect is due to integrin αvβ3 and downstream pathway FAK-PI3K/Akt-NF-κB; therefore, inhibitors of these elements could provide tools for pathology where this signaling due to MCP-1 is compromised such as diabetic retinopathy (You et al., 2014).

The signal transduction pathway of the effects of thyroid hormones in THP-1 monocytes was studied by a pharmacological approach, and we found an involvement of both PI3K and MAPK pathway in the inhibition of cell migration induced by MCP-1 and IGF-1. In particular, wortmannin, an inhibitor of PI3K pathway, prevented both MCP-1 and IGF-1 stimulation of THP-1 monocyte migration, as expected, and MAPK pathway inhibition with PD98059 prevented thyroid hormone inhibition of migration induced by IGF-1, as already reported for different cells and different responses, such as glucose uptake (Incerpi et al., 2014), but not that induced by MCP-1. On the contrary, our data show that thyroid hormones did not act through MAPK pathway, or at least not only, to block cell migration induced by MCP-1, since in the presence of PD98059, the inhibition of migration by T_4_ was still partly present, suggesting that other pathways could be involved. As to PI3K, this pathway is important for the stimulation of cell migration by both MCP-1 and IGF-1. Wortmannin did not recover the inhibition of cell migration induced by thyroid hormone on either cytokine, and the cell migration was practically at the basal level when wortmannin was present, with some difference in the presence of MCP-1.

We also studied the long-term thyroid hormone modulation of IGF-1-stimulated proliferation in THP-1 monocytes. Again, T_4_ inhibited the proliferation stimulated by IGF-1, and the effect was mediated by MAPK pathway, since PD98059 prevented the inhibition by T_4_ of the IGF-1-induced cell proliferation. RGD peptide blocked the effect of T_4_, demonstrating the involvement of the integrin αvβ3, in agreement with the migration experiments. Again, the analogy with the behavior of thyroid hormone, T_4_, in L-6 myoblasts is striking: a long-term effect mediated by integrin αvβ3 starts at the plasma membrane, one more example of crosstalk between non-genomic and genomic long-term responses (Cheng et al., 2010; Incerpi et al., 2014). We found the same behavior of T_4_ in THP-1 monocytes for the cell proliferation induced by MCP-1. T4 inhibited the stimulation of proliferation by MCP-1, and in the presence of RGD, the effect of T_4_ on the proliferation was not reverted but eventually potentiated.

Chemokines such as MCP-1 mediate their effects through a receptor combined to a G-protein, the downstream effects are not so well defined. In any case, there is an increase of cAMP, PI3K activation, and an increase of tyrosine phosphorylation leading to the activation of STAT-1α, a member of the Signal Transducers and Activators of Transcription family, and actin polymerization. This implies also the activation of the MAPK pathway leading to the NO increase (Biswas et al., 2001). STAT-1α plays a key role in the upregulation of IFN-regulated genes involved in the innate immune response. Thyroid hormone, as well as IGF-1 and MCP-1, was able to stimulate STAT-1α in 30 min, as already shown in different cell lines (Lin et al., 1999a; Shih et al., 2004), with a decreased effect up to 2 h and an increase again at 4 h. T_4_ prevented STAT-1α tyrosine phosphorylation induced by IGF-1 at 2 and 4 h of stimulation. Taken together, these results lead us to hypothesize that maybe two different mechanisms could be involved in this process, or the same mechanism starting from the plasma membrane and going to the nucleus, both non-genomic and genomic leading to a production of cytokines and chemokines for a more sustained humoral immune response, given by the activation of transcription factors downstream STAT leading to inflammation through the synthesis of chemokines and cytokines but also cytotoxicity, prosurvival signaling (Lin et al., 1999a, b).

Results of migration experiments suggest a role of NO, since L-NAME, an NO inhibitor, was able to revert the stimulatory effect of MCP-1 in THP-1 cell migration. On the other hand, T_4_ inhibits the stimulatory effect of GSNO in THP-1 cell migration. Thyroid hormones inhibit NO by upregulation of GSH (Deb and Das, 2011). In addition to this, a crosstalk between integrin αvβ3 and NADPH oxidase has been suggested (De Vito et al., 2012; Gnocchi et al., 2012). NADPH oxidase gives rise to ROS that impair or uncouple NOS function, resulting in a decrease of NO production. This situation reported also for amyloid-β vascular dysfunction and other pathologies could explain the lack of NO increase both in the fluorescent and in the Griess assays (Lamoke et al., 2015).

Baicalein inhibits the inflammation stimulated by LPS by inhibiting the expression of cytokines and chemokines such as MCP-1 and Toll-like receptor 4 (TLR4)/nuclear factor κB (NF-κB) pathway in human umbilical vein endothelial cells (HUVECs). Baicalein inhibits also NO and radicals production in microglia activated by LPS (Li et al., 2005; Wan et al., 2018). Chen et al. (2012) showed that thyroid hormone, in a dose-dependent way, promoted NO production through iNOS stimulation after meningococcal infection of murine macrophage cell line RAW 264.7 and human THP-1 macrophages. It might be that the experimental conditions used in our laboratory, lack of infection, did not allow enough NO production to be detected. NO is involved in the crosstalk between thyroxine and T-lymphoma cells. In fact, after long-term treatment with thyroxine, there is an induction of apoptosis of T lymphoma cells through an increase of oxidative stress species from iNOS activity (Barreiro Arcos et al., 2013). The increase of ROS by NADPH oxidase and mitochondrial ROS activated the first and NO production by cytokines and downstream signaling including PI3K–Akt axis, but also the MAPK pathway and STAT-1α activation are among the first steps leading to Nod-like receptor protein 3 (NLRP3) inflammasome activation, giving rise to physiological responses aimed for bactericidal clearance, cell survival, and an anti-inflammatory condition (De Luca et al., 2021).

*In silico* molecular docking simulations carried out in order to better understand the mechanism of interaction between T_4_ and αvβ3 integrin in its inactive form are in agreement with those obtained by the migration assays that show a significant potentiation in the inhibition of the migration induced by MCP-1, when T_4_ was in the presence of the peptide RGD. As mentioned above, this effect could be due to the presence on the integrin of a binding site for the hormone, different from the RGD site.

## Conclusion

Thyroid hormones in our experimental system behave as anti-inflammatory agents both in the presence of MCP-1 and IGF-1 by a different mechanism, resulting in a stronger inhibitory effect in the presence of MCP-1 with respect to IGF-1. We can hypothesize that the role of thyroid hormones as anti-inflammatory agents may be different depending on the physiopathological situation.

## Data Availability Statement

The original contributions generated for this study are included in the article/supplementary material, further inquiries can be directed to the corresponding authors.

## Author Contributions

EC, SI, RDL, PDV, PJD, H-YL, EA, FP, and JZP: conceptualization of work and writing of the manuscript, editing, and formatting manuscript and figures. RM, MM, ZP, and PDV: performing experiments. PDV: editing of the manuscript. MB and RG: organization of experiments of cytokine assay and editing of the manuscript. TP and MC: nitric oxide experiments and editing and discussing the manuscript. FP: docking experiments, editing, and discussion on the manuscript. All authors read and approved the final form of the manuscript.

## Conflict of Interest

The authors declare that the research was conducted in the absence of any commercial or financial relationships that could be construed as a potential conflict of interest. The handling editor declared a past co-authorship with one of the authors RDL.
